# Music and Technology: The Curative Algorithm

**DOI:** 10.3389/fpsyg.2017.02055

**Published:** 2017-11-24

**Authors:** Alfredo Raglio, Francisco Vico

**Affiliations:** ^1^Music Therapy Laboratory, Istituti Clinici Scientifici Maugeri, Pavia, Italy; ^2^ETSI Informatica, Andalucia Tech, University of Malaga, Malaga, Spain

**Keywords:** music listening, therapy, artificial intelligence, Melomics-Health, scientific method

The power of music listening lies principally in its substantial impact on the brain (Zatorre, 2003; Peretz and Zatorre, 2005; Koelsch, 2009, 2011, 2014, 2015; Zatorre and Salimpoor, 2013; Koelsch et al., 2015), resulting in important psycho-physiological repercussions (Boso et al., 2006; Loomba et al., 2012; Chanda and Levitin, 2013; Koelsch and Jäncke, 2015; Perrone-Capano et al., 2017). From its very beginnings, music has served diverse aims (Figure 1): on the one hand it provides a vehicle for expression, pleasure and aesthetic fruition, on the other it accompanies many events of social, religious or other nature. In the latter case, the use of music can be defined as functional, or as serving a particular purpose. This results in specific musical characteristics which support the musical function: examples are lullabies, work songs, religious ceremonies and so on. Besides these aspects, music has always encompassed a therapeutic value as well. This perspective has gained recognition through the centuries, supporting the identification of the scientific foundations of music's therapeutic action (Hillecke et al., 2005; Koelsch, 2009). The resultant applications in numerous clinical, rehabilitative contexts have led to important therapeutic results (Hole et al., 2015; Lee, 2016; McConnell et al., 2016; Raglio et al., 2016; Zhao et al., 2016; Geretsegger et al., 2017; Magee et al., 2017; van der Steen et al., 2017). A previous study (Chamorro-Premuzic and Furnham, 2007) discussed about different functions and uses of music in people's live linking them also to personality and cognitive ability.

**Figure 1:**
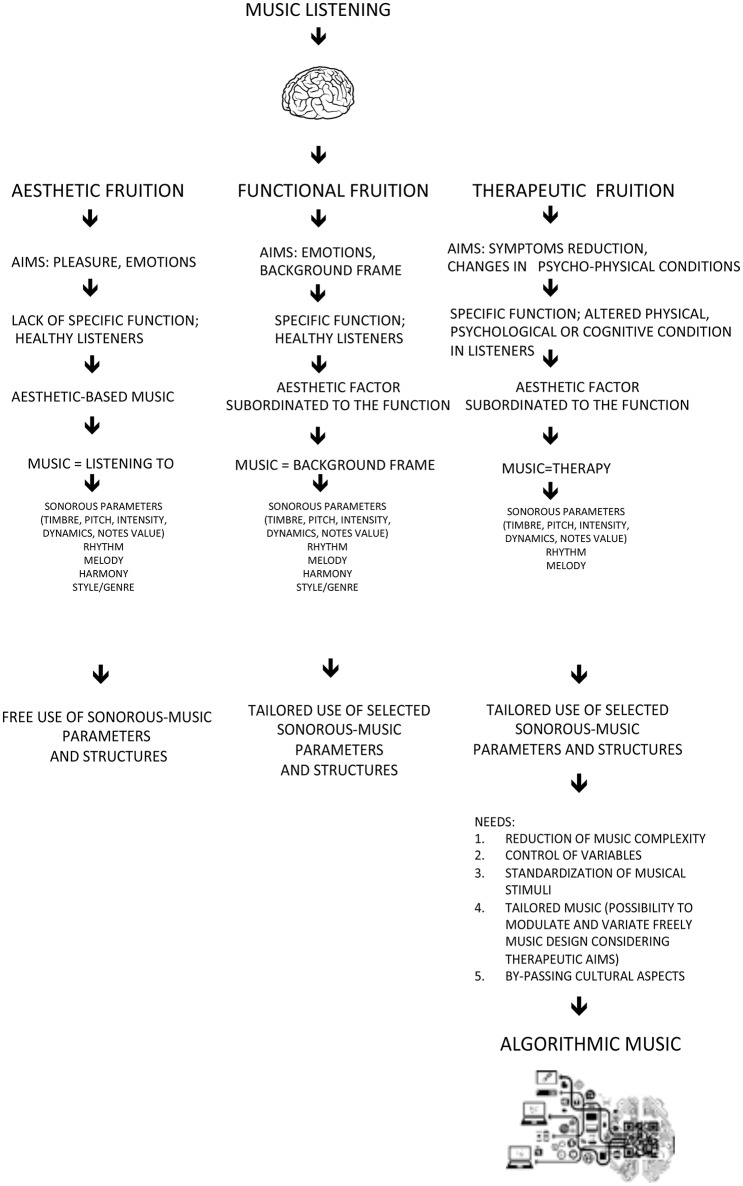
Music listening: functions, aims and music structures.

Analysing the ways in which music listening is used, aesthetic fruition can be attributed to the condition of searching for pleasure and emotional expression, without a specific goal. In this case the music is based on aesthetic components which create styles/types of music, which change with the times and have always been profoundly affected by the culture of the location in which they develop. Music meant for a specific fruition is generally based on the same cultural norms, but is governed by restrictions and musical rules which take into account not only the aesthetic factor but also the purpose the music is to serve. This has resulted in the composition of music which is effective in specific contexts, due to structural and parametric qualities and their effect on the individual. This explains for example why lullabies share common characteristics which make them similar in all cultures, because the aim of the music (not purely aesthetic) requires the restriction of compositional models characterized by specific criteria regarding form, parameters and expressive styles, which facilitate the achievement of the determined aim. Such restrictions are even more evident in music created for therapeutic aims, that is with the objective of reducing a symptom or modifying the psycho-physical condition of the beneficiary.

Today, within the context of the evolution of music and its compositional forms, coinciding with scientific development, we are in a situation of impasse, given that the complexity of music does not easily correspond with the scientific need to relate musical/sound phenomena with their potential therapeutic efficacy. Not surprisingly, a recent meta-analysis (Hole et al., 2015) concludes that despite some methodological and clinical questions as yet unanswered, music produces significant effects during surgery, even though it is not yet clear which type of music should be proposed and which processes support the effects (pleasure? Distraction? Something else?). It is our opinion that research should aim to bridge these gaps, certainly not an easy task, maybe starting from some precise considerations on music.

If music is therapeutic, we should be able to affirm which music carries this attribute, in which clinical conditions, with which modes of administration, and why. It is also important to understand if the effect of the music is direct or indirect, in other words, if for example other similar situations of pleasure or distraction produce analogous results. Much research is still needed to answer these points, but we believe that it is important to give a new direction and perspective to these studies.

It is our opinion that artificial intelligence could contribute significantly in this sense. Artificial intelligence has already been employed to produce music according to a particular style (Fernandez and Vico, 2013). Recently both Iamus and Melomics (Ball, 2012; Fernandez and Vico, 2013; Requena et al., 2014) have created respectively aleatory (contemporary style) and pop music, by introducing an algorithm capable of producing music in this sense without however referring to pre-existing composers or styles.

Melomics-Health (see Supplementary Material for an audio file example and related score—Supplementary Figure 1), a new algorithm derived from Melomics, is currently being tested to create relaxing music for therapy, in particular for temporary symptoms which characterize frequently clinical situations (i.e., pre-surgical anxiety, stress, pain, etc.).

The Melomics-Health compositions are made as a sequence of fragments, each one being defined independently with the following parameters:

- Timbre: name of the instrument
- Pitch range: lowest and highest possible notes from within the instrument's tessitura (pitches cover the range from C_0_ to B_7_)
- Time signature: 3/4, 3/8, 2/4, 4/4…
- Key: as a key signature. Key signatures are named with the name of the tonic, followed by the minus or plus symbol for minor or major scales, and there are the notes corresponding to the scale of each tonality. Notes are named in scientific pitch notation (better known as international pitch notation), with a letter from A to G, followed by the sharp or flat symbol (if it applies). When the note is referred to a concrete octave, the number of the octave follows as a subscript.
- Tempo: bpm = beats per minutes (equivalent to adagio, andante, allegro, etc.)
- Intervals: range of allowed pitch difference between notes (2-3, 2-4, etc.)
- Rhythm: density of note durations (as a probability for each duration)
- Dynamics: “p” for piano, “mf” for mezzoforte, etc.
- Duration: overall duration of the fragment in seconds

In order to create the musical structure of each fragment, its overall duration is divided in a number of beats, as given by the tempo. In a way, it is like creating a grid where all possible note durations (from the rhythm parameter) can fit, and then filling in the bins with concrete notes by roulette wheel selection (Goldberg, 1989) (Supplementary Figure 2). This algorithm selects the notes according to their probabilities. Once the duration of the note has been chosen, then it is given a pitch, according to the pitch of the previous note (starting on an average pitch from the pitch range parameter) and to the allowed intervals.

The score is written in Lilypond notation, so each parameter of the fragment is translated to this scripting language, in a way that the score can be printed and synthesized to determine its validity for the given therapeutic use, and its correction.

This algorithmic process does not negate the value of music: rather it effects a simplification, reducing music to its essence and thus leading to a better control of its structure and parameters.

This allows us, on the basis of clinical experience and literature, to hypothesize music with specific characteristics aimed for therapeutic use: the music's structure and its parameters can be controlled, modeled and adapted to the therapeutic needs. Thus music becomes a therapeutic mediator conceived specifically for this end and therefore free of cultural references belonging to musical styles or genres.

The music of Melomics-Health is based on the use of the western musical scale but not on usual harmonic and melodic structures/connections. This allows to recognize Melomics-Health compositions as music that we can consider a-temporal and free from specific emotional or cognitive references. A music which tends to retrieve an archaic condition, returning to the origins of musical material in an encounter with the demands of science. Science in fact sets the objective of verifying the effectiveness of such musical material based on precise knowledge of its integral elements, with the possibility (given by technology) of shaping these as desired in accordance with therapeutic needs. Any given existing music poses the problem of complexity resulting from the co-existence of parameters and structures occurring within a pre-defined form, which cannot be modified without distorting the music itself. As an alternative to this, we believe that artificial intelligence can contribute the possibility to create musical compositions with specific characteristics modifiable according to therapeutic demands, combining art and technology and benefiting the encounter between music and science (Figure 1).

A possible limitation of Melomics-Health music is that it does not take into account musical patterns and repetition as usually it can be found in music. However we think that our auditory and cognitive systems can gradually recognized the structure underlying Melomics-Health compositions making that music familiar and partially predictable. On the other hand repetition and predictability of music are related to liking and reward aspects but we are not sure that other much important effects in the therapeutic field (i.e., activation, relaxation, modulation, etc.) are mainly based on those factors.

In this regard Melomics-Health approach not only defines new therapeutic perspectives, but also prepares the ground for innovative models of neuroscience research on the impact of music/sound phenomena on the human brain.

## Author contributions

AR: Study concept, music and music therapy expertise and article writing; FV: Study concept, IT expertise and article writing. Both Authors approved the final version of the article.

### Conflict of interest statement

The authors declare that the research was conducted in the absence of any commercial or financial relationships that could be construed as a potential conflict of interest.
