# Supplementary material for: Music and Technology: The Curative Algorithm
Source: Front Psychol. 2017 Nov 24;8:2055. doi: 10.3389/fpsyg.2017.02055 (PMC5715368; doi:10.3389/fpsyg.2017.02055)
Supplement: Supplementary file 1 [file DataSheet1.PDF]

Supplementary Material

Music and Technology: the curative algorithm

Alfredo Raglio*, Francisco Vico

*** Correspondence:** Corresponding Author: alfredo.raglio@icsmaugeri.it

Supplementary Data

Audio file. Example from Melomics-Health composition excerpt. The pre-defined sonorous-music parameters of this kind of song were thought to compose relaxing music to use in stressful clinical conditions (i.e. pre-operative anxiety, chronic pain, etc).

Supplementary Figures

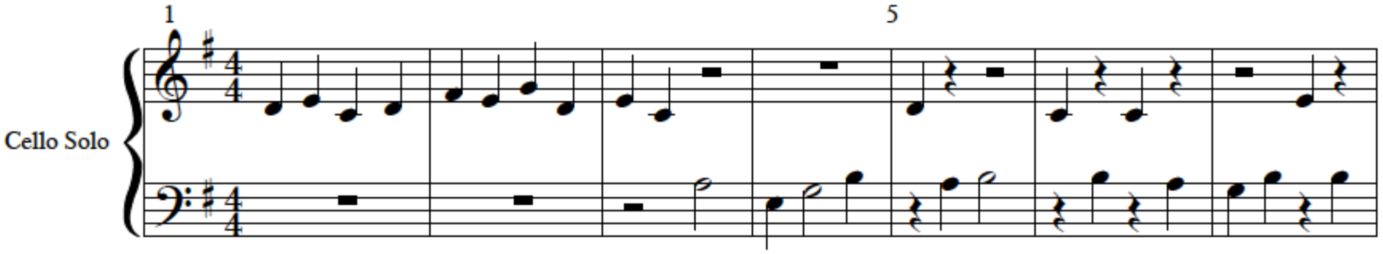

Supplementary Figure 1. Score related to Melomics-Health composition excerpt (Audio file)

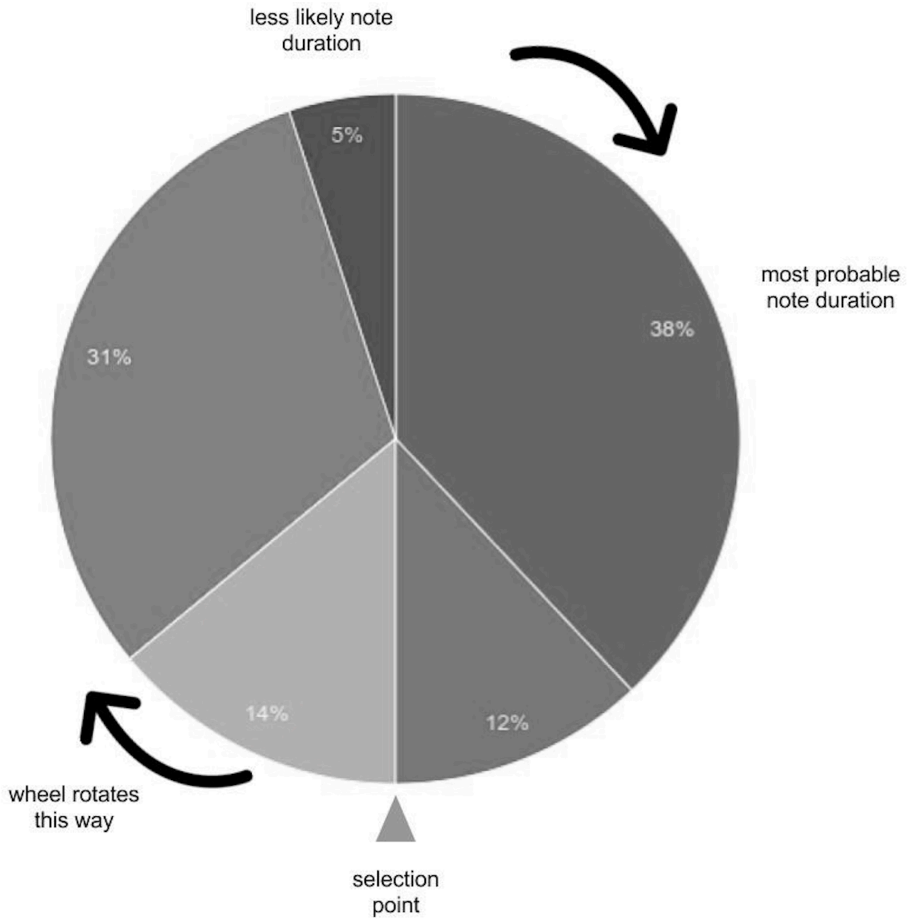

Supplementary Figure 2. Schematic representation of the roulette wheel selection, where each note duration is given a probability of occurrence and the wheel, as it spins, selects more notes from those durations that are more likely to occur.
